# Functional outcomes and biochemical control after radical prostatectomy versus irreversible electroporation for localized prostate cancer

**DOI:** 10.1016/j.isci.2026.117303

**Published:** 2026-09-09

**Authors:** Zhi-Yu Xia, Jia-Cheng Xiang, Yu-Xuan Yang, Fan Xiao, Jun Yang, Shao-Gang Wang, Qi-Dong Xia

**Affiliations:** 1 Department of Urology, Tongji Hospital, Tongji Medical College, Huazhong University of Science and Technology, Wuhan, China

**Keywords:** prostate cancer, erectile function, urinary continence, radical prostatectomy, irreversible electroporation

## Abstract

Treatment selection for localized prostate cancer requires balancing oncologic control against urinary and sexual function. We retrospectively compared 32 men undergoing laparoscopic bilateral nerve-sparing radical prostatectomy (RP) with 18 undergoing focal irreversible electroporation (IRE), using unweighted and inverse probability of treatment weighting analyses. IRE was associated with shorter operative time and less frequent early urinary incontinence and sexual function decline. At 12 months after weighting, urinary incontinence occurred in 6.0% after RP and 0% after IRE, while sexual function decline occurred in 40.0% and 4.1%, respectively. Biochemical recurrence was more frequent after IRE than RP at 12 months (28.6% versus 8.0%). These findings suggest a trade-off between early functional preservation and biochemical control and should be interpreted as hypothesis-generating because of the retrospective single-center design, small sample, non-equivalent recurrence definitions, and short follow-up.

## Highlights

• IRE was associated with less frequent early urinary incontinence than RP
• IRE was associated with less frequent sexual function decline than RP
• RP showed better early biochemical control than IRE at 12 months
• The findings support individualized counseling for localized prostate cancer

## Graphical abstract

**Figure:**
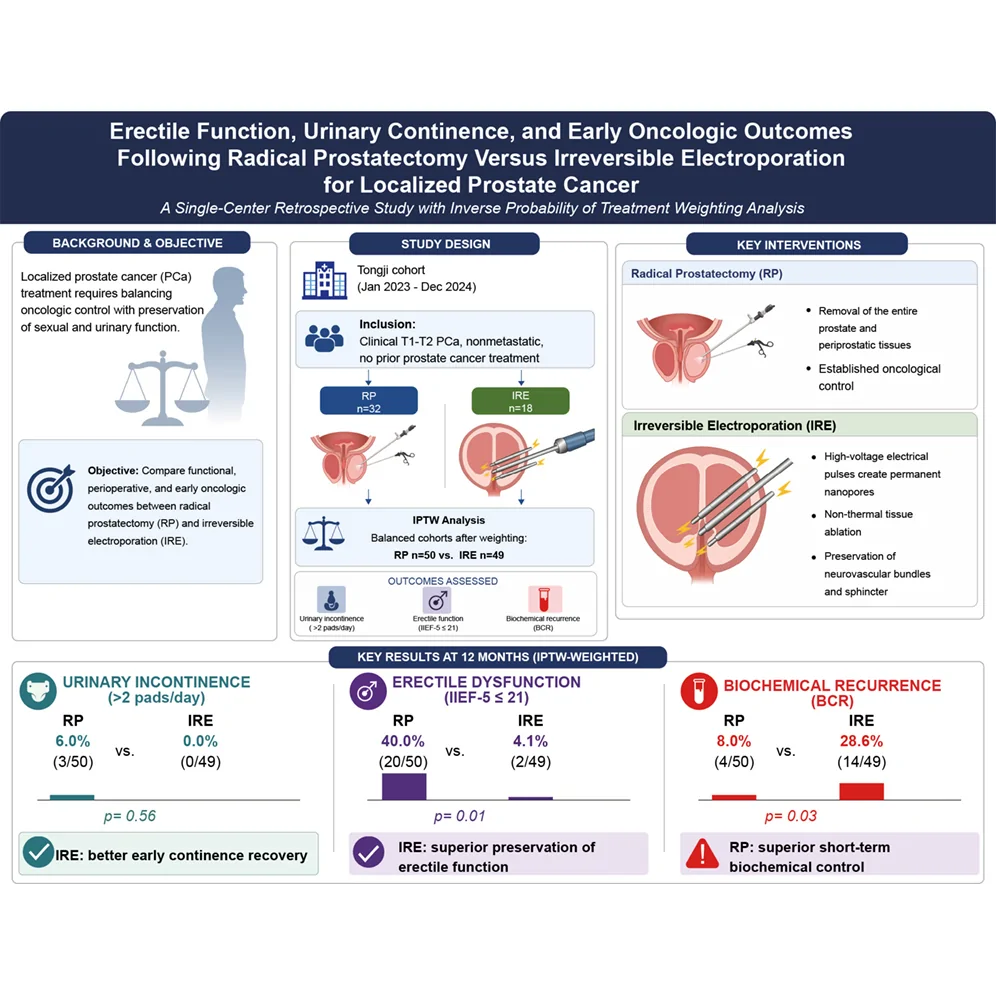

## Teaser

Health sciences; Medicine; Medical specialty; Internal medicine; Urology

## Introduction

Prostate cancer (PCa) is the second most frequently diagnosed malignancy among men worldwide and remains a leading cause of cancer-related morbidity and mortality.^1^^,^^2^^,^^3^ With the widespread implementation of prostate-specific antigen (PSA) screening and advances in diagnostic imaging, an increasing proportion of patients are diagnosed at a clinically localized stage.^4^^,^^5^ Given the generally favorable survival outcomes of localized PCa, preservation of health-related quality of life, particularly urinary continence and sexual function, has become an increasingly important consideration in treatment decision-making.^6^ For these patients, management strategies range from active surveillance to definitive local therapies such as surgery and radiotherapy. Selecting the optimal treatment, therefore, requires careful consideration of both oncologic efficacy and treatment-related functional outcomes. However, balancing oncologic control with preservation of sexual and urinary function remains a major challenge in clinical practice.

Radical prostatectomy (RP) has long been regarded as the cornerstone of curative-intent treatment for localized PCa.^4^^,^^7^^,^^8^ Long-term follow-up studies have demonstrated durable oncologic control and favorable survival outcomes following RP. However, these benefits are often accompanied by substantial functional impairment. Postoperative urinary incontinence and erectile dysfunction are common complications and may persist long term, significantly affecting patients’ quality of life. Because these sequelae strongly influence long-term survivorship and treatment satisfaction, they are highly relevant to contemporary andrological practice. Despite advances in surgical technique and the widespread adoption of robotic-assisted approaches, functional recovery after RP remains highly variable.

In response to these limitations, focal therapy has emerged as an alternative treatment paradigm aimed at minimizing treatment-related morbidity while maintaining acceptable oncologic control in selected patients.^5^^,^^9^ Irreversible electroporation (IRE) is a relatively novel, non-thermal ablative technology that induces permanent nanopore formation in cell membranes through the delivery of high-voltage electrical pulses, ultimately leading to apoptotic cell death.^10^^,^^11^^,^^12^ Importantly, IRE preserves the extracellular matrix architecture, including collagenous structures, blood vessels, and neural pathways. Because IRE is non-thermal and preserves extracellular matrix architecture, it may reduce collateral injury to structures relevant to erectile function and urinary continence compared with whole-gland surgery. This distinctive mechanism has generated interest in its application for PCa, where preservation of the neurovascular bundles and urethral sphincter is critical for maintaining urinary continence and sexual function. RP represents an established whole-gland definitive treatment, whereas IRE is a focal, function-preserving strategy intended to ablate the index lesion while minimizing injury to adjacent functional structures.

Despite increasing clinical adoption, the role of IRE in PCa treatment remains incompletely defined.^9^^,^^11^^,^^13^^,^^14^^,^^15^ Current guideline statements remain cautious regarding focal therapy, including IRE, because robust long-term comparative evidence remains limited; focal ablative approaches should therefore be discussed within a careful patient-selection framework and, where possible, in clinical trial or registry settings.^16^ RP and IRE differ in treatment intent, eligibility, and evidence base; therefore, they should not be viewed as competing options for all patients with localized PCa. Existing studies are often limited by small sample sizes, heterogeneous patient selection, and relatively short follow-up durations. Moreover, direct comparative data between IRE and RP are scarce, particularly in real-world Asian clinical setting. The present study aimed to compare sexual and urinary functional outcomes, together with perioperative and early oncologic outcomes, between RP and IRE in a single-center cohort, with both unweighted and inverse probability of treatment weighting (IPTW) analyses to minimize selection bias.

## Results

### Baseline characteristics

**Figure 1:**
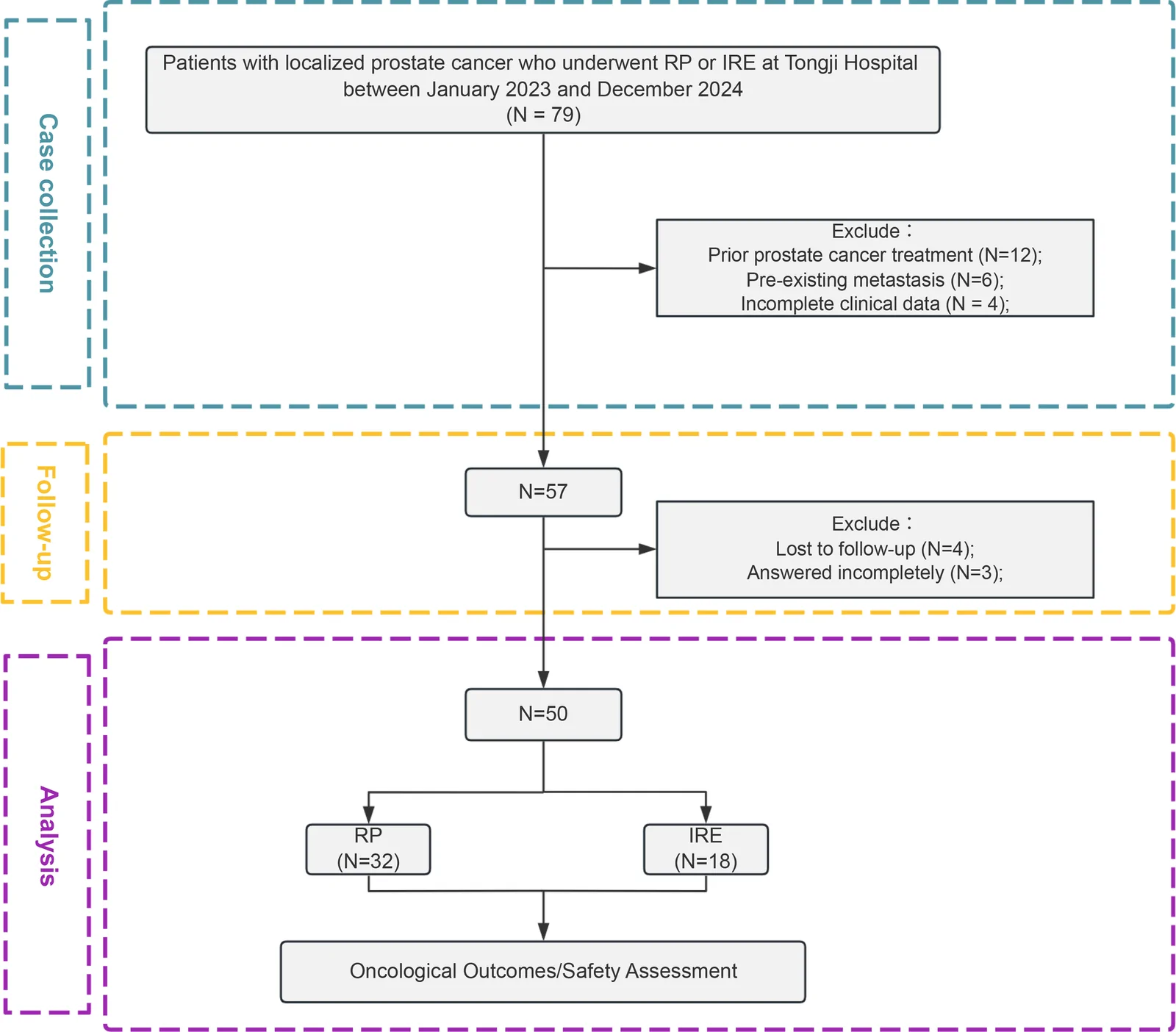
Flow diagram of patient selection and study cohort

A total of 50 patients met the inclusion criteria, including 32 treated with RP and 18 treated with IRE (Figure 1). Baseline characteristics before and after IPTW are summarized in Table 1. After IPTW, the weighted pseudo-population estimates were RP *n* = 50 and IRE *n* = 49. Before weighting, PSA level and Gleason score showed meaningful between-group imbalance, consistent with real-world treatment selection. After IPTW adjustment, baseline covariate balance improved across measured variables. Table 1 also reports clinical T stage, Prostate Imaging Reporting and Data System (PI-RADS) score, and baseline 5-item International Index of Erectile Function (IIEF-5). All patients in both groups had cT2 disease. PI-RADS distributions were comparable after weighting (weighted *p* = 0.84, Standardized Mean Difference (SMD) = 0.17). Baseline IIEF-5 was also comparable after weighting—23.0 (21.9–24.0) vs. 22.0 (22.0–23.0), weighted *p* = 0.79, SMD = 0.08.

**Table 1:** Baseline characteristics of patients undergoing radical prostatectomy or irreversible electroporation before and after inverse probability of treatment weighting

	Unweighted RP (*n* = 32)	Unweighted IRE (*n* = 18)	*p* value	SMD	Weighted RP (*n* = 50)	Weighted IRE (*n* = 49)	*p* value		SMD
**Age**									

mean (SD), y	67.3 (7.0)	65.1 (8.4)	0.31	0.30	66.5 (7.0)	66.7 (8.8)	0.95		0.02
≤60	18.7% (6)	27.8% (5)	0.46	–	24.0% (12)	22.4% (11)	0.86		–
>60	81.2% (26)	72.2% (13)	–	–	76.0% (38)	77.5% (38)	–		–

**BMI**									

mean (SD)	24.6 (3.4)	24.1 (2.8)	0.55	0.18	24.2 (3.3)	23.8 (3.0)	0.68		0.13

**PSA**									

median (IQR), ng/mL	13.1 (7.2–17.4)	9.0 (6.3–13.2)	0.07	0.57	11.9 (6.8–16.5)	12.2 (8.2–14.7)	0.65		0.13
≤10	34.3% (11)	55.5% (10)	0.14	–	58.0% (29)	57.1% (28)	0.93		–
>10	65.6% (21)	44.4% (8)	–	–	42.0% (21)	42.9% (21)	–		–

**Gleason scores**									

≤6	40.6% (13)	66.7% (12)	0.08	0.60	14.0% (7)	14.3% (7)	0.94		0.01
7	59.4% (19)	33.3% (6)	–	–	86.0% (43)	85.7% (42)	–		–

**Risk group**									

low	18.7% (6)	38.9% (7)	0.12	0.46	24.0% (12)	26.5% (13)	0.92		0.01
intermediate	81.3% (26)	61.1% (11)	–	–	76.0% (38)	73.5% (36)	–		–

**PI-RADS score**									

2	6.2% (2)	5.6% (1)	0.79	0.29	9.1% (4)	9.0% (4)	0.84		0.17
3	15.6% (5)	22.2% (4)	–	–	18.0% (9)	14.2% (7)	–		–
4	56.2% (18)	61.1% (11)	–	–	55.4% (28)	63.5% (31)	–		–
5	21.9% (7)	11.1% (2)	–	–	17.5% (9)	14.2% (7)	–		–

**IIEF-5**									

median (IQR)	22.5 (21.8–23.2)	22.0 (22.0–23.0)	0.84	0.16	23.0 (21.9–24.0)	22.0 (22.0–23.0)	0.79		0.08
≤21	25.0% (8)	16.7% (3)	0.72	–	24.1% (12)	15.4% (8)	–		–
>21	75.0% (24)	83.3% (15)	–	–	75.9% (38)	84.6% (41)		–	–

**Clinical T stage**									

cT2	32/32 (100.0)	18/18 (100.0)	1.00	0.00	50/50 (100.0)	49/49 (100.0)		1.00	0.00

### Perioperative outcomes

Perioperative outcomes are presented in Table 2. The median operative time was significantly shorter for IRE compared with RP (80 min vs. 201 min, *p* < 0.001). The length of hospital stay did not differ significantly between groups, with a median of 6.0 days for IRE and 5.0 days for RP (*p* = 0.41). Severe perioperative complications were defined as Clavien-Dindo grade ≥ III, and no such events were observed in either group.

**Table 2:** Perioperative outcomes of patients treated with radical prostatectomy or irreversible electroporation

	RP (*n* = 32)	IRE (*n* = 18)	*p* value
Follow-up (months)	21.0 (18.0–23.0)	18.5 (15.7–22.0)	0.12
Operative time (min)	201 (170–242)	80 (69–92)	<0.001
Length of hospital stay (d)	5.0 (5.0–7.0)	6.0 (5.0-7.3)	0.41

### Oncologic outcomes

**Figure 2:**
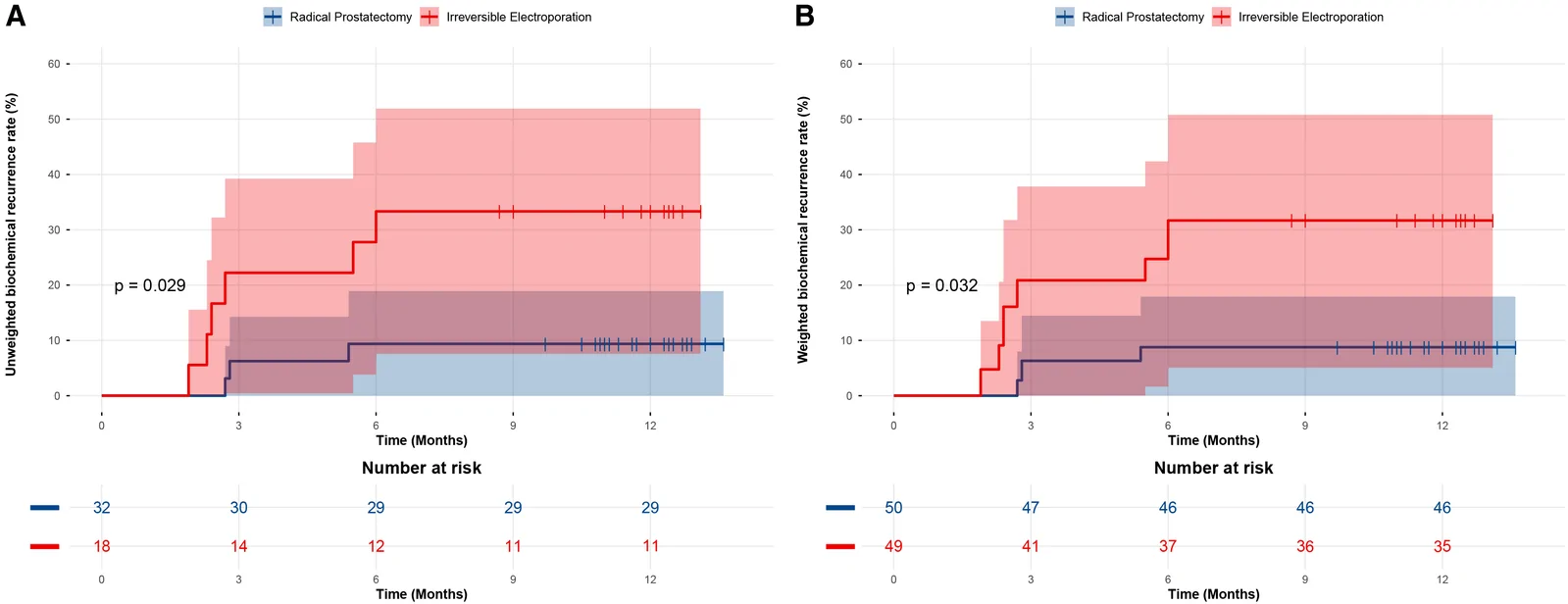
Kaplan-Meier estimates of cumulative biochemical recurrence after radical prostatectomy and irreversible electroporation (A) Unweighted analysis (RP *n* = 32; IRE *n* = 18; log-rank *p* = 0.029); (B) IPTW-weighted analysis (RP weighted *n* = 50; IRE weighted *n* = 49; logrank *p* = 0.032). Numbers at risk and 95% confidence intervals are shown. The weighted sample sizes are pseudo-population estimates rather than actual patient counts. These curves describe early biochemical outcomes and should not be interpreted as evidence of durable long-term oncologic superiority.

Oncologic and functional outcomes after RP and IRE in both unweighted and IPTW-weighted analyses are summarized in Table 3. At the 1-month follow-up, no biochemical recurrence (BCR) events were observed in either treatment group. Differences in oncologic outcomes emerged during follow-up. At 3 months, BCR occurred in 22.2% of patients in the IRE group and 6.3% in the RP group, although this difference did not reach statistical significance (*p* = 0.17). At 6 months, BCR occurred in 33.4% of patients after IRE and 9.4% after RP in the unweighted analysis (*p* = 0.05) and in 24.5% versus 8.0% in the IPTW-weighted analysis (*p* = 0.06). At 12 months, BCR occurred in 9.4% of patients after RP versus 38.9% after IRE in the unweighted analysis (*p* = 0.02) and 8.0% versus 28.6% in the IPTW-weighted analysis (*p* = 0.03). Given the median follow-up of 21.0 months after RP and 18.5 months after IRE, these oncologic findings should be interpreted as early biochemical outcomes rather than evidence of durable long-term oncologic superiority. Kaplan-Meier analysis showed a difference in early cumulative BCR between groups (Figure 2).

**Table 3:** Oncologic and functional outcomes after radical prostatectomy versus irreversible electroporation in unweighted and IPTW-weighted analyses

	Unweighted RP (*n* = 32)	Unweighted IRE (*n* = 18)	*p* value	Weighted RP (*n* = 50)	Weighted IRE (*n* = 49)	*p* value
**Biochemical recurrence (%)**						

1-month follow-up	0/32 (0)	0/18 (0)	1.00	0/50 (0.0)	0/49 (0.0)	1.00
3-month follow-up	2/32 (6.3)	4/18 (22.2)	0.17	3/50 (6.0)	8/49 (16.3)	0.19
6-month follow-up	3/32 (9.4)	6/18 (33.4)	0.05	4/50 (8.0)	12/49 (24.5)	0.06
12-month follow-up	3/32 (9.4)	7/18 (38.9)	0.02	4/50 (8.0)	14/49 (28.6)	0.03

**Urinary incontinence (%)**						

1-month follow-up	28/32 (87.5)	3/18 (16.7)	<0.001	45/50 (90.0)	7/49 (14.3)	<0.001
3-month follow-up	9/32 (28.1)	1/18 (5.6)	0.07	16/50 (32.0)	2/49 (4.1)	0.01
6-month follow-up	7/32 (21.9)	1/18 (5.6)	0.23	11/50 (22.0)	2/49 (4.1)	0.05
12-month follow-up	2/32 (6.3)	0/18 (0)	0.53	3/50 (6.0)	0/49 (0.0)	0.56

**Decline in sexual function (%)**						

1-month follow-up	20/32 (62.5)	2/18 (11.1)	<0.001	28/50 (56.0)	7/49 (14.3)	0.02
3-month follow-up	17/32 (53.1)	2/18 (11.1)	0.01	24/50 (48.0)	7/49 (14.3)	0.05
6-month follow-up	15/32 (46.9)	1/18 (5.6)	<0.001	22/50 (44.0)	2/49 (4.1)	<0.001
12-month follow-up	14/32 (43.7)	1/18 (5.6)	<0.001	20/50 (40.0)	2/49 (4.1)	0.01

### Functional outcomes

#### Urinary continence

**Figure 3:**
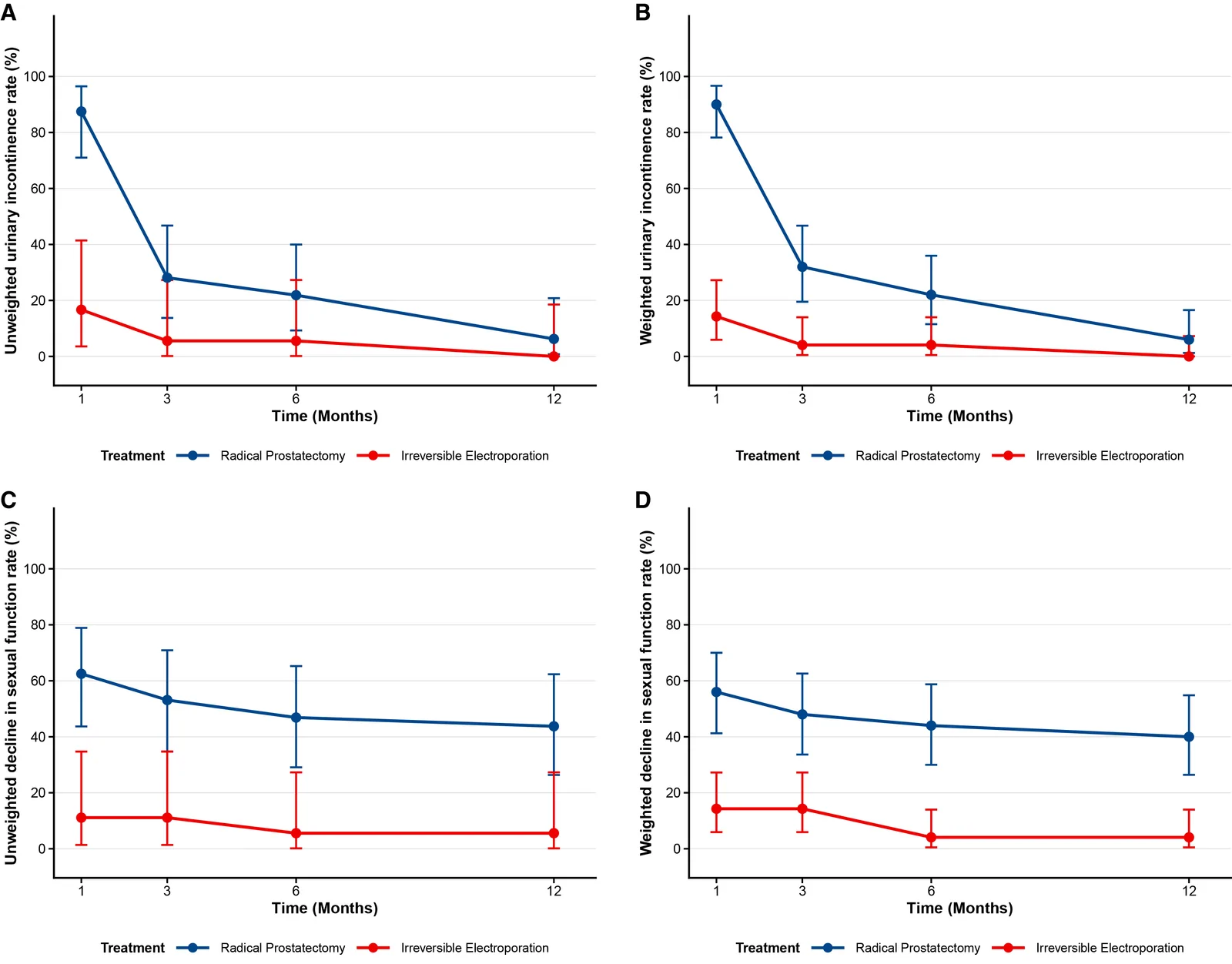
Functional outcomes during follow-up after radical prostatectomy versus irreversible electroporation (A) Unweighted urinary incontinence rate (RP *n* = 32; IRE *n* = 18). (B) IPTW-weighted urinary incontinence rate (RP weighted *n* = 50; IRE weighted *n* = 49). (C) unweighted sexual function decline rate (RP *n* = 32; IRE *n* = 18). (D) IPTW-weighted sexual function decline rate (RP weighted *n* = 50; IRE weighted *n* = 49). Error bars indicate 95% confidence intervals. Urinary incontinence was defined as use of at least two pads per day, and sexual function decline was defined as an IIEF-5 decrease of more than 4 points from baseline. Weighted sample sizes are pseudo-population estimates rather than actual patient counts. No significance asterisks are used in this figure; exact time point *p* values are reported in Table 3. PCa, prostate cancer; IRE, irreversible electroporation; RP, radical prostatectomy; PSA, prostate-specific antigen; csPCa, clinically significant prostate cancer; SD, standard deviation; IQR, interquartile range; IPTW, inverse probability of treatment weighting; mpMRI, multiparametric MRI.

Urinary incontinence rates were lower among patients treated with IRE during early follow-up. In the unweighted analysis, 87.5% of patients treated with RP required at least two pads per day at 1 month, compared with only 16.7% in the IRE group (*p* < 0.001). The IPTW-weighted analysis yielded consistent results (90.0% vs. 14.3%, *p* < 0.001). Although continence improved over time in both groups, the RP cohort continued to show higher rates of incontinence during early follow-up. By 12 months, the proportion of patients requiring at least two pads per day was low and comparable between groups in both analyses (6.3% vs. 0% unweighted, *p* = 0.53; 6.0% vs. 0% weighted, *p* = 0.56) (Figures 3A and 3B). A sensitivity analysis using 0 pads/day or 0–1 safety pad/day was not feasible because the retrospective records did not reliably distinguish a single safety pad from true leakage.

#### Sexual function decline

Sexual function was assessed using IIEF-5 scores,^17^ and sexual function decline was defined as a decrease of more than 4 points from baseline. In Table 1, baseline IIEF-5 scores were similar between groups before weighting, 22.5 (21.8–23.2) vs. 22.0 (22.0–23.0), *p* = 0.84, SMD = 0.16, and remained balanced after IPTW, 23.0 (21.9–24.0) vs. 22.0 (22.0–23.0), weighted *p* = 0.79, SMD = 0.08. At 1, 3, 6, and 12 months, sexual function decline occurred in 62.5%, 53.1%, 46.9%, and 43.7% of RP patients and 11.1%, 11.1%, 5.6%, and 5.6% of IRE patients, respectively (*p* < 0.001, *p* = 0.01, *p* < 0.001, and *p* < 0.001, respectively) (Table 3; Figures 3C and 3D).

## Discussion

In this single-center retrospective study, we compared RP and IRE in men with localized PCa, with particular attention to sexual function decline and urinary continence. Our findings suggest a clinically meaningful trade-off between short-term oncologic control and preservation of sexual and urinary function. Compared with RP, IRE was associated with less frequent sexual function decline, less early urinary incontinence, shorter operative time, and consistent results on both unweighted and IPTW-weighted analyses.^15^^,^^18^^,^^19^ In contrast, RP was associated with a lower 12-month BCR rate and lower cumulative recurrence during early follow-up. Accordingly, the present comparison should be interpreted as a patient-counseling framework for balancing whole-gland oncologic control against focal treatment-related functional preservation rather than as evidence that these modalities are interchangeable for all patients. These findings are particularly relevant to andrological practice because treatment-related changes in sexual function and continence often play a central role in treatment decision-making for men with localized PCa.

The lower BCR rate observed after RP is consistent with prior literature demonstrating durable oncologic control following whole-gland radical surgery.^6^^,^^7^^,^^8^ By removing the entire prostate gland along with periprostatic tissues, RP reduces the risk of residual microscopic disease and untreated satellite lesions, which are common in multifocal PCa.^20^ In contrast, focal IRE targets the index lesion and a limited surrounding margin, and untreated clinically significant disease outside the ablation zone remains a recognized cause of residual disease or early recurrence.^14^^,^^15^^,^^18^ Because residual benign and potentially malignant prostate tissue remains after focal IRE, PSA kinetics and PSA-based BCR after IRE are not directly equivalent to BCR after RP. The apparent difference in early cumulative BCR should therefore be interpreted cautiously because the RP and IRE recurrence definitions reflect different post-treatment anatomy and PSA kinetics.^21^^,^^22^

In addition to disease multifocality, accurate delineation of tumor margins remains a critical limitation for IRE. Although multiparametric MRI has improved lesion localization and treatment planning, MRI can underestimate true tumor extent and may miss clinically significant lesions, particularly in small-volume or anterior disease.^23^ Notably, multicenter validation data suggest that multiparametric MRI (mpMRI) alone is insufficient to exclude residual disease after IRE, underscoring the importance of systematic follow-up biopsy regardless of MRI findings.^24^

Despite these oncologic concerns, the early functional differences observed in this study were substantial. Patients treated with IRE had less frequent early urinary incontinence and sexual function decline than those treated with RP, consistent with the tissue-sparing mechanism of non-thermal electroporation and prior IRE series.^11^^,^^13^^,^^15^^,^^18^^,^^19^ These findings may be relevant for carefully selected patients, although the retrospective design precludes causal interpretation.

From an andrological perspective, these findings may be especially meaningful. Men considering definitive treatment for localized PCa are often asked to weigh cancer control against the risks of erectile dysfunction and urinary leakage. In clinical practice, these functional consequences may influence treatment choice as strongly as oncologic considerations, particularly in patients with low- or intermediate-risk disease. Our data, therefore, support a more individualized counseling framework in which expected sexual and urinary outcomes are discussed explicitly alongside oncologic risk. In this context, IRE may be considered for carefully selected men who prioritize preservation of sexual function and early continence recovery, provided that they understand the possibility of closer surveillance and potentially less favorable short-term biochemical outcomes.^15^^,^^16^^,^^21^ These results, therefore, apply only to carefully selected patients and should not be generalized to all men with localized PCa.

Operative time was shorter in the IRE group. However, this study did not directly assess anesthesia-related morbidity, postoperative recovery time, or outcomes in older or comorbid subgroups; therefore, the clinical consequences of the shorter procedure cannot be determined from these data. Emerging studies have examined the immune effects of IRE, but these mechanisms were not evaluated in this cohort and do not affect the interpretation of the present clinical findings.^25^

Nevertheless, the clinical application of IRE must be approached with caution. Patients should be carefully counseled regarding the increased risk of early BCR and the need for rigorous post-treatment surveillance, including repeat biopsy when appropriate. Patients treated with IRE require close surveillance, including serial PSA testing, mpMRI follow-up when suspicious, and repeat targeted and/or systematic biopsy when biochemical or imaging findings suggest residual or recurrent clinically significant cancer.^21^^,^^23^^,^^24^ Randomized and registry data indicate that out-of-field disease remains common even when focal and extended IRE achieve similar early oncologic outcomes, reinforcing the importance of meticulous patient selection and mapping biopsies.^14^^,^^18^

### Limitations of the study

Several limitations of the present study should be considered when interpreting these findings. First, this was a retrospective, single-center study with a relatively modest sample size (*n* = 50), which inherently limits statistical power and generalizability. Second, treatment allocation was not randomized. Although IPTW was performed based on propensity scores derived from age, body mass index (BMI), preoperative PSA, Gleason score, National Comprehensive Cancer Network (NCCN) risk group, PI-RADS score, and baseline IIEF-5 score to balance measured baseline confounders, residual confounding from unmeasured variables cannot be entirely excluded. Because of the modest cohort size, IPTW estimates should be interpreted cautiously even after assessment of overlap, weight distribution, covariate balance, and effective sample size. Third, the follow-up duration was relatively short, and oncologic assessment was limited to early BCR rather than longer-term endpoints such as metastasis-free or cancer-specific survival. Finally, although erectile function was evaluated using the validated IIEF-5 questionnaire, other functional outcomes may still have been affected by the inherent limitations of retrospective data collection. Additional limitations include potential IPTW instability in a small cohort, selection bias related to treatment allocation, non-equivalent BCR definitions after RP and IRE, lack of uniform protocol-mandated post-IRE biopsy surveillance, and the inability to perform a continence sensitivity analysis using 0 pads/day or 0–1 safety pad/day because raw pad-use categories were not reliably captured. Although all RP procedures used a laparoscopic bilateral nerve-sparing approach and all IRE procedures were performed as focal ablation, these procedural differences should be considered when interpreting functional outcomes. Use of phosphodiesterase type 5 inhibitors and structured erectile rehabilitation was not consistently recorded, which may have influenced postoperative IIEF-5 recovery. Clinical T stage and PI-RADS score were available for analysis; however, other potentially relevant clinical variables, including detailed lesion location, positive biopsy cores, percentage core involvement, tumor volume, and PSA density, were not consistently available in the retrospective dataset and therefore could not be included in the baseline adjustment or outcome analyses. Validated urinary quality-of-life questionnaires were not collected, limiting assessment of urinary bother, urgency, and broader patient-reported urinary outcomes. Time-to-continence recovery could not be reliably assessed because exact recovery dates were not consistently captured.

These limitations notwithstanding, the study adds comparative real-world data on functional and early oncologic outcomes after RP and IRE in a Han Chinese cohort. Future research should prioritize prospective multicenter studies with larger sample sizes, standardized treatment allocation criteria, and longer follow-up. In addition, more granular assessment of sexual recovery, continence recovery, and post-IRE surveillance outcomes would help clarify which patients are most likely to benefit from function-preserving focal therapy.^13^^,^^15^^,^^18^^,^^19^ Beyond clinical and imaging variables, emerging molecular biomarkers may further refine individualized treatment selection and surveillance by capturing tumor heterogeneity, therapeutic resistance, and prognostic risk; however, molecular biomarkers were not evaluated in the present cohort and should be investigated in future studies.^26^ A stronger evidence base is needed before IRE can be positioned more clearly within treatment pathways for men who seek both oncologic safety and preservation of sexual and urinary function.

In this comparative analysis using both unweighted and IPTW-adjusted methods, IRE was associated with lower early urinary incontinence and less frequent sexual function decline, whereas RP provided better short-term biochemical control. These findings highlight the importance of individualized treatment selection for men with localized PCa. IRE may represent a promising function-preserving option for carefully selected patients who prioritize urinary and sexual functional preservation and accept close surveillance, whereas RP remains the more established approach for patients prioritizing oncologic control.

## Resource availability

### Lead contact

Further information and requests should be directed to and will be fulfilled by the lead contact, Qi-Dong Xia (qidongxia_md@163.com).

### Materials availability

This study did not generate new unique reagents.

### Data and code availability

• Aggregate data supporting the findings are included in the article; Tables 1, 2, and 3; and supplemental information. De-identified participant-level clinical data are not publicly deposited because unrestricted release of this small single-center clinical cohort could increase participant re-identification risk and is restricted by applicable ethics and institutional data-governance requirements. Requests may be directed to the lead contact and will be considered on a case-by-case basis, subject to a scientifically justified proposal, approval by the relevant ethics committee and institutional data-governance body, and completion of an appropriate data-use agreement. No public accession code is available for participant-level data.
• The R scripts used for IPTW analyses and figure generation are not publicly deposited. Access to analysis code may be requested from the lead contact and will be considered under the same controlled-access conditions as the participant-level data. No public accession code is available for the analysis code.
• This paper does not report newly generated reagents, plasmids, cell lines, protein structures, sequencing datasets, or microscopy/western blot source images. Software, clinical resources, and controlled-access items are listed in the key resources table.

## Acknowledgments

The authors thank all patients who participated in this study. This research received no specific grant from any funding agency in the public, commercial, or not-for-profit sectors.

## Author contributions

Z.-Y.X., J.-C.X., and Y.-X.Y. contributed to study conception and design, data collection, data analysis, and drafting of the manuscript. F.X. contributed to data collection and literature review. J.Y., S.-G.W., and Q.-D.X. contributed to supervision of the study, interpretation of data, and critical revision of the manuscript. All authors read and approved the final manuscript.

## Declaration of interests

The authors declare no conflicts of interest.

## STAR★Methods

### Key resources table

**Table:** 

REAGENT or RESOURCE	SOURCE	IDENTIFIER
**Deposited data**		

Aggregate study data and IPTW diagnostics	This paper	Tables 1, 2, 3, S1, and S2
De-identified participant-level clinical data	This paper	Restricted access; requests to the lead contact require ethics and institutional approval and a data-use agreement

**Software and algorithms**		

IBM SPSS Statistics	IBM	Version 26.0; RRID:SCR_002865
R	R Foundation for Statistical Computing	Version 4.3.2; RRID:SCR_001905; https://www.r-project.org/
R scripts for IPTW analyses and figure generation	This paper	Not publicly deposited; controlled access through the lead contact subject to approval

**Other**		

International Index of Erectile Function-5 questionnaire	Rosen et al.^17^	https://doi.org/10.1038/sj.ijir.3900472
REMD G1 compound steep pulse therapeutic apparatus	Shanghai Nuosheng Medical Technology Co., Ltd.	Model HFMP-01
Disposable ablation electrodes	Shanghai Nuosheng Medical Technology Co., Ltd.	Used with the HFMP-01 apparatus; catalog number not available

### Experimental model and study participant details

#### Study design and participants

We conducted a single-center retrospective cohort study of patients with clinically localized prostate cancer who underwent RP or IRE at the Department of Urology, Tongji Hospital, Tongji Medical College, Huazhong University of Science and Technology, between January 2023 and December 2024. The study was conducted in accordance with the Declaration of Helsinki and was approved by the Institutional Ethics Review Committee of Tongji Hospital (approval no. TJ-IRB202409097). The committee waived the requirement for individual informed consent. All participants were adult Han Chinese men; their age distribution is reported in Table 1. Because the cohort included men with prostate cancer only, sex- or gender-stratified comparisons were not performed. This retrospective observational study was not a clinical trial and was not prospectively registered.

Eligible patients had histologically confirmed prostatic adenocarcinoma, clinical T1-T2 disease, and no evidence of lymph-node involvement or distant metastasis. Patients with previous prostate cancer treatment, including radiotherapy or focal ablation, were excluded. Patients with incomplete clinical data or insufficient follow-up were also excluded.

### Method details

#### Treatment selection

Treatment selection was based on shared decision-making after review of tumor risk, lesion localization, technical suitability for focal ablation, expected functional trade-offs, physician recommendation, and patient preference; no formal multidisciplinary-team allocation process was used. Before treatment selection, all patients underwent clinical assessment, PSA testing, mpMRI, histologic confirmation by prostate biopsy, clinical staging, and NCCN risk stratification. Before IRE, patients underwent mpMRI for lesion localization and biopsy-based assessment, including transperineal systematic biopsy and MRI-informed targeted biopsy when a suspicious lesion was identified.

IRE was considered when nonmetastatic, clinically localized disease was suitable for lesion-directed focal ablation, the dominant lesion could be localized on mpMRI and biopsy, and preservation of urinary continence and sexual function was a major treatment priority after counseling. Under the device instructions and institutional selection framework, IRE was primarily considered for adults with low- or intermediate-risk localized prostate cancer, PSA <20 ng/mL, clinical T1a-T2c disease, Gleason score ≤7, no lymph-node or distant metastasis, and expected survival longer than 5 years. RP was selected when whole-gland treatment was preferred or considered more appropriate because of broader disease extent, unfavorable lesion location for focal ablation, concern for multifocal or poorly localized disease, patient preference, or physician recommendation after counseling.

#### Treatment procedures

RP was performed using a laparoscopic bilateral nerve-sparing approach. All IRE procedures were performed as lesion-directed focal ablation using the REMD G1 compound steep pulse therapeutic apparatus (HFMP-01) and disposable ablation electrodes. The planned ablation field targeted the index lesion and extended at least 5 mm beyond the visible tumor margin, with an overall ablation boundary of approximately 5–10 mm when anatomically feasible. Treatment planning was individualized according to lesion location, prostate anatomy, mpMRI findings, biopsy localization, and ultrasound-guided transperineal electrode placement. Electrode exposure length was selected from 5 to 40 mm according to tumor size, inter-electrode distance was approximately 0.5-2.0 cm, and ablation was delivered between electrode pairs using compound steep pulses.

#### Data collection and variables

Baseline demographic and clinical variables were extracted from electronic medical records, including age, ethnicity, BMI, preoperative PSA, Gleason score, NCCN risk group, clinical T stage, PI-RADS score, and baseline IIEF-5 score. Detailed lesion location, positive biopsy cores, percentage core involvement, tumor volume, and PSA density were not consistently available. Baseline urinary continence was reviewed when available. Use of phosphodiesterase type 5 inhibitors and structured erectile rehabilitation after treatment was not consistently recorded. Perioperative variables included operative time and length of postoperative hospital stay.

#### Follow-up and outcome definitions

Patients were followed after treatment, with PSA measured at 1, 3, 6, and 12 months. Post-IRE surveillance included serial PSA measurement at scheduled visits, mpMRI when clinically suspicious, and repeat biopsy when PSA kinetics or imaging suggested residual or recurrent disease. A uniform protocol-mandated post-IRE biopsy was not performed. BCR was defined as PSA ≥0.2 ng/mL after RP or a PSA increase ≥2.0 ng/mL above the post-treatment nadir after IRE.^21^^,^^22^

Urinary incontinence was defined as use of at least two pads per day because the retrospective records did not reliably distinguish a single safety pad from true leakage. Sexual function was evaluated using the IIEF-5 questionnaire at baseline and during follow-up. Questionnaires were administered during scheduled follow-up visits and retrospectively extracted from the medical records. Sexual function decline was defined as a decrease of more than 4 points from baseline. IIEF-5 scores range from 5 to 25, with higher scores indicating better erectile function.^17^

### Quantification and statistical analysis

Continuous variables were summarized as mean ± standard deviation or median (interquartile range) and compared using Student’s *t* test or the Mann-Whitney U test. Categorical variables were summarized as counts and percentages and compared using the chi-square test or Fisher’s exact test. Time to BCR was estimated using the Kaplan-Meier method and compared using the log rank test. Figure 3 displays 95% confidence intervals for functional-outcome proportions; no significance asterisks are used.

To address measured baseline differences, propensity scores were estimated using logistic regression including age, BMI, PSA, Gleason score, NCCN risk group, PI-RADS score, and baseline IIEF-5 score. Unstabilized average-treatment-effect IPTW weights were used without weight trimming. Balance was assessed using standardized mean differences, with an absolute SMD <0.20 considered acceptable. IPTW-adjusted sample sizes are weighted pseudo-population estimates rather than actual patient counts. Propensity-score overlap, weight distribution, covariate balance, and effective sample size diagnostics are reported in the supplemental information. All analyses were conducted using unweighted and IPTW-weighted data. A two-sided *p* < 0.05 was considered statistically significant. Analyses were performed using SPSS version 26.0 and R version 4.3.2. Given the modest cohort and sparse events, weighted estimates were interpreted cautiously and exploratory multivariable models were not used for definitive inference.

## Supplemental information

Document S1. Figures S1–S3

Table S1. Covariate balance diagnostics before and after inverse probability of treatment weighting

Table S2. Weight diagnostics and effective sample size for the inverse probability of treatment weighting analysis
