# Supplementary material for: Functional outcomes and biochemical control after radical prostatectomy versus irreversible electroporation for localized prostate cancer
Source: iScience. 2026 Sep 9;29(9):117303. doi: 10.1016/j.isci.2026.117303 (PMC13581978; doi:10.1016/j.isci.2026.117303)
Supplement: Supplementary file 1 [file mmc1.pdf]

iScience, Volume *29*

Supplemental information

Functional outcomes and biochemical control after radical prostatectomy versus irreversible electroporation for localized prostate cancer

Zhi-Yu Xia, Jia-Cheng Xiang, Yu-Xuan Yang, Fan Xiao, Jun Yang, Shao-Gang Wang, and Qi-Dong Xia

Supplementary Figure S1. Propensity score overlap

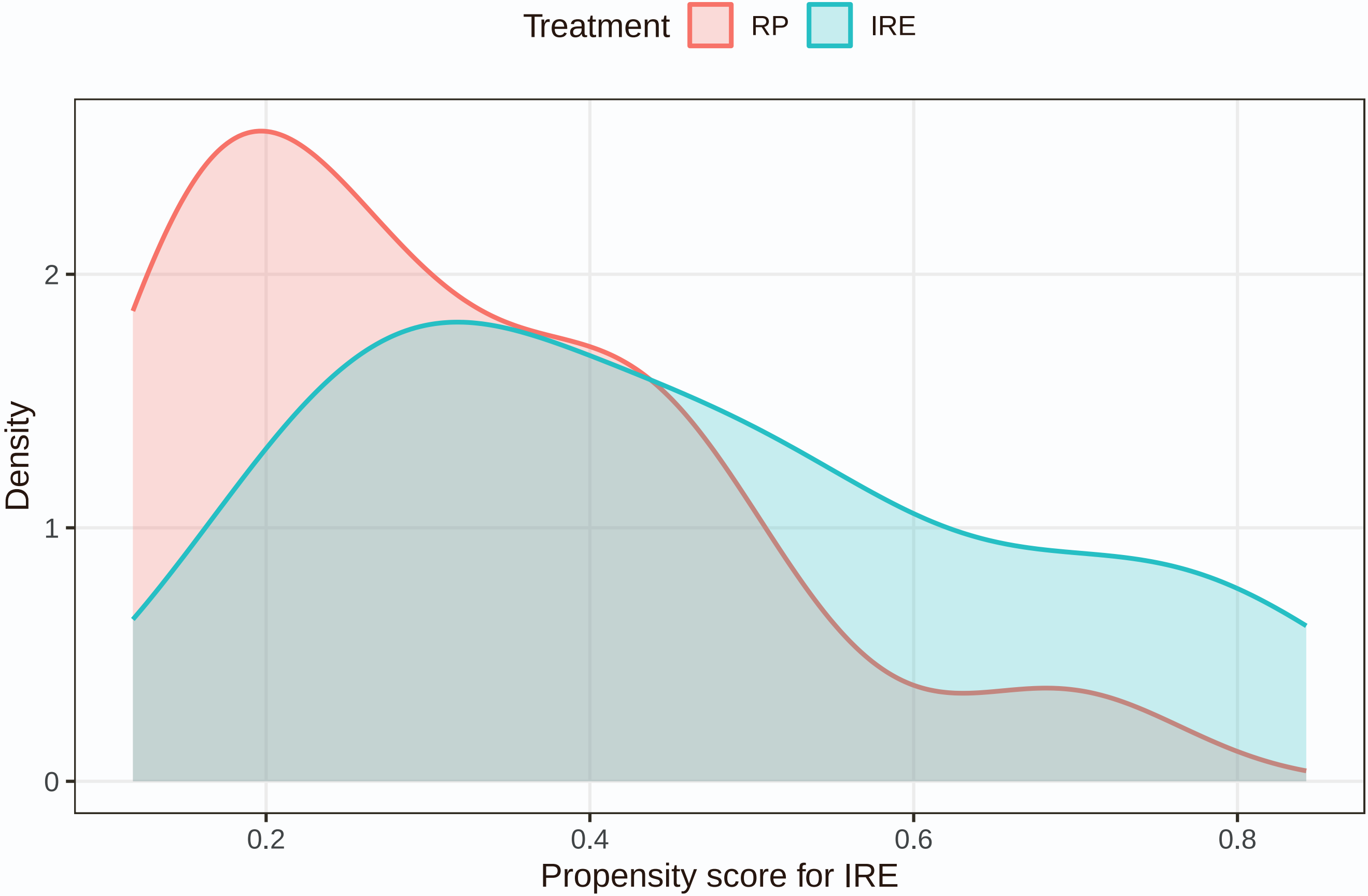

Supplementary Figure S2. Distribution of IPTW weights

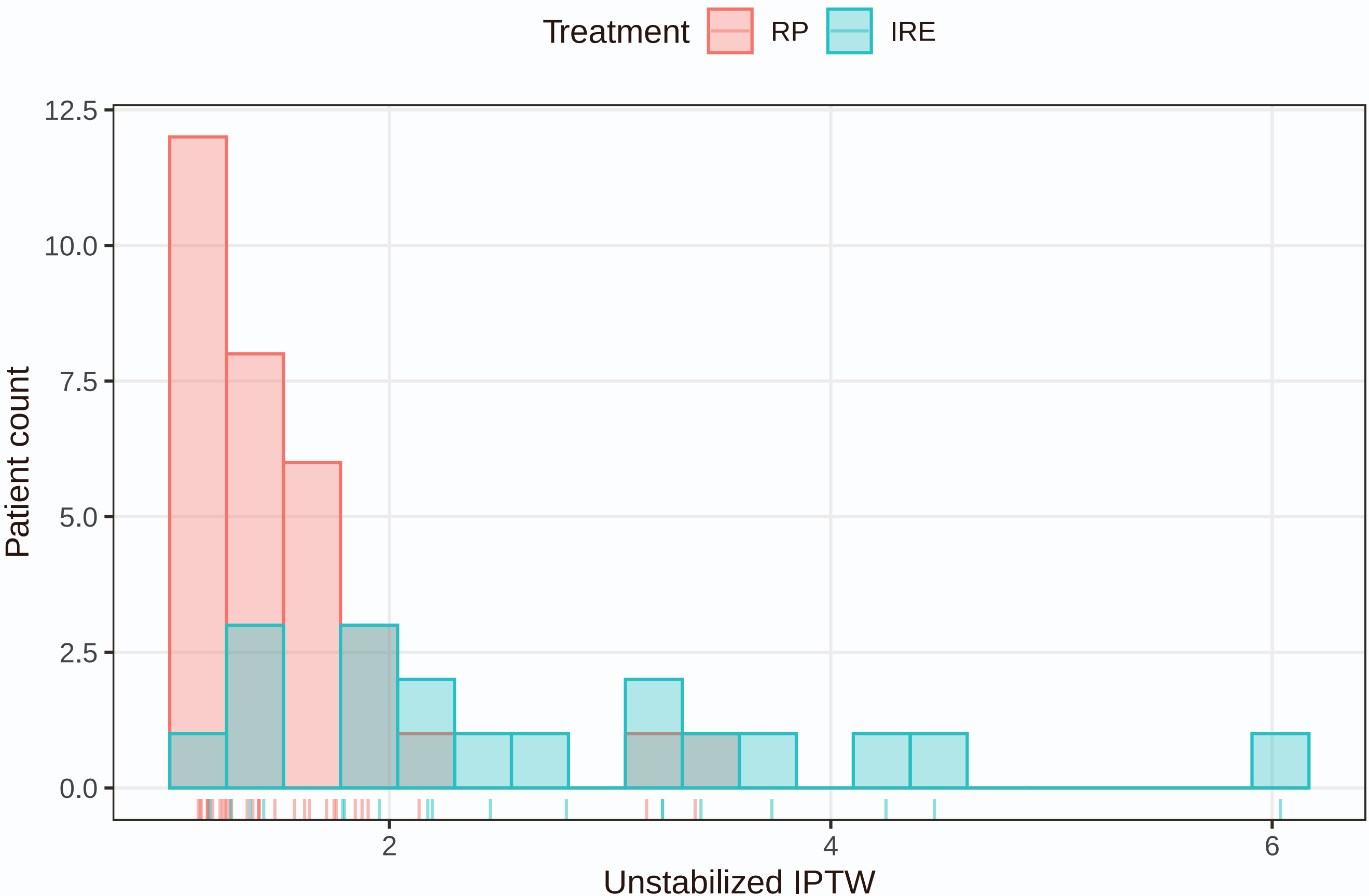

Supplementary Figure S3. Covariate balance before and after IPTW

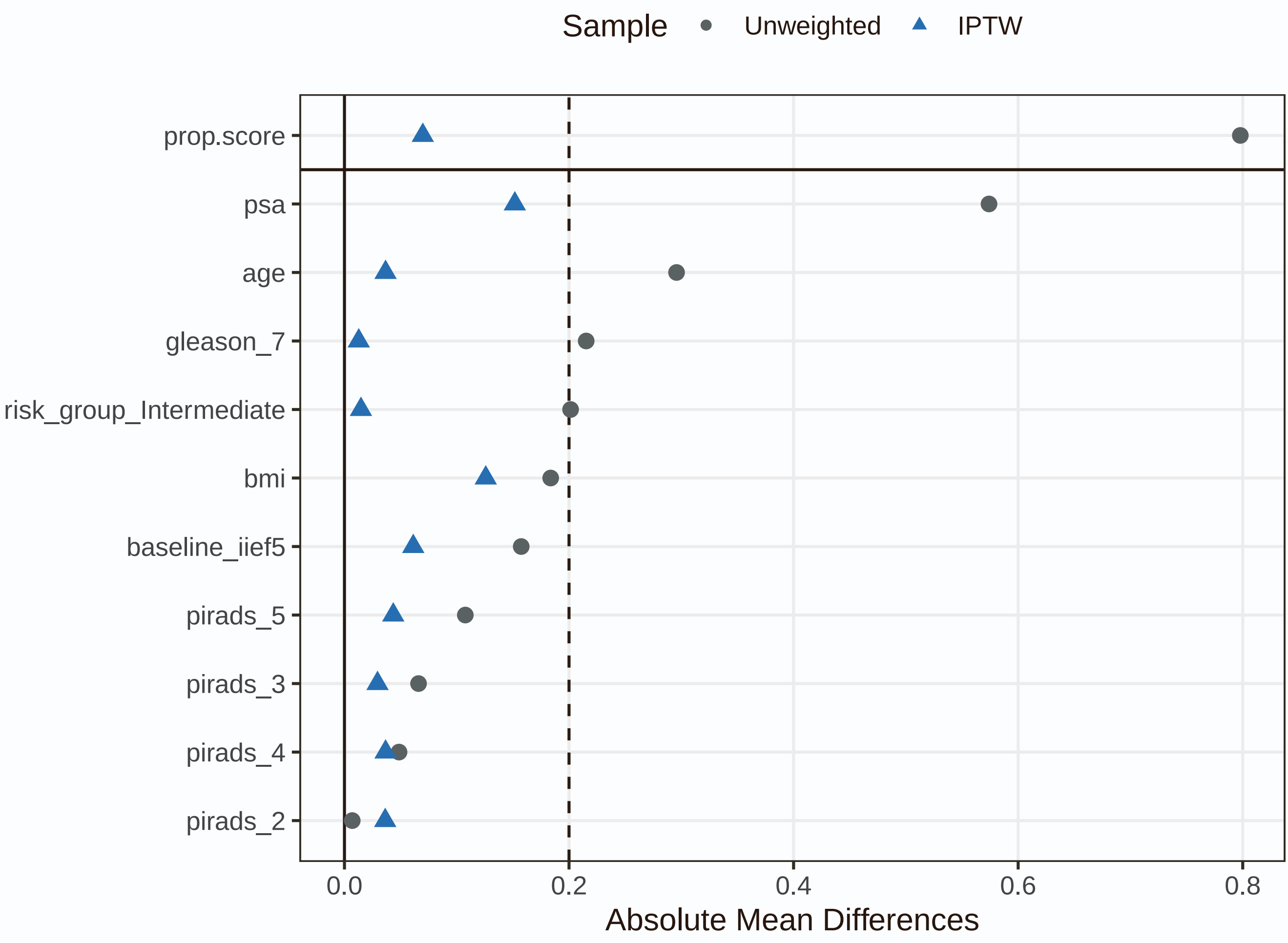

Supplementary Figure Legends

Supplementary Figure S1. Propensity-score overlap between treatment groups. Density distributions of the estimated propensity scores for receiving irreversible electroporation (IRE) are shown for the radical prostatectomy (RP) and IRE groups. Propensity scores were estimated using a logistic regression model including age, body mass index, prostate-specific antigen, Gleason score, NCCN risk group, PI-RADS score, and baseline IIEF-5 score. The plot demonstrates partial but not perfect overlap between groups, consistent with real-world treatment selection.

Supplementary Figure S2. Distribution of inverse probability of treatment weights. Histogram and rug plot showing the distribution of unstabilized inverse probability of treatment weights (IPTW) by treatment group. The maximum IPTW weights were 3.39 in the RP group and 6.04 in the IRE group. This diagnostic plot was used to evaluate the presence of influential weights in the weighted analysis.

Supplementary Figure S3. Covariate balance before and after IPTW. Love plot showing absolute standardized mean differences for measured baseline covariates before and after IPTW. After weighting, all adjusted absolute standardized mean differences were below 0.20, with the largest adjusted absolute standardized mean difference observed for prostate-specific antigen (0.152). The propensity-score standardized mean difference improved from 0.798 before weighting to 0.070 after weighting.

Abbreviations: BMI, body mass index; IIEF-5, International Index of Erectile Function-5; IPTW, inverse probability of treatment weighting; IRE, irreversible electroporation; NCCN, National Comprehensive Cancer Network; PI-RADS, Prostate Imaging Reporting and Data System; PSA, prostate-specific antigen; RP, radical prostatectomy; SMD, standardized mean difference.
